# Satisfaction with cosmesis and priorities for cosmesis design reported by lower limb amputees in the United Kingdom: Instrument development and results

**DOI:** 10.1177/0309364613512149

**Published:** 2014-12

**Authors:** Nicola Cairns, Kevin Murray, Jonathan Corney, Angus McFadyen

**Affiliations:** 1 Department of Design, Manufacture and Engineering Management, University of Strathclyde, Glasgow, UK; 2 National Centre for Prosthetics and Orthotics, Department of Biomedical Engineering, University of Strathclyde, Glasgow, UK; 3 AKM-STATs, Statistical Consultant, Glasgow, UK

**Keywords:** Amputation, questionnaire, cosmesis, transfemoral, transtibial, lower limb, satisfaction, importance, prosthetic design

## Abstract

**Background:**

Amputee satisfaction with cosmesis and the importance they place on cosmesis design have not been published in the literature.

**Objectives:**

To investigate the current satisfaction levels of amputees in the United Kingdom with their cosmesis and the importance placed on attributes of cosmesis design to inform future cosmesis redesign.

**Study Design:**

Cross-sectional questionnaire study.

**Methods:**

Questionnaires were administered to lower limb amputees in the United Kingdom. Satisfaction scores and the overall importance ranking of cosmesis features were calculated. Statistically significant relationships between two demographic, satisfaction or importance variables were tested using Fisher’s exact tests (one-tailed) at a significance level *p* = 0.05.

**Results:**

Between 49% and 64% of respondents reported neutral or dissatisfied opinions with the cosmesis features (greater than 50% for five of the nine features). The three most important features identified were shape matching the cosmesis to the sound limb, free prosthetic joint movement underneath the cosmesis and natural fit of clothing over the cosmesis.

**Conclusions:**

The results indicate that current cosmesis satisfaction levels of amputees in the United Kingdom are below what the medical device industry and clinical community would desire. The most important cosmesis features identified by the sample can be used to direct future cosmesis design research.

**Clinical relevance:**

The findings will enable the medical device industry to improve cosmesis design in the areas that are important to amputees. The findings also counter anecdotal opinions held by clinicians, providing an opportunity for them to evaluate any preconceptions they harbour and how this might influence their clinical work.

## Background

Limb amputation can lead to a negative change in how an individual mentally perceives their own body, known as body image,^1^ as they attempt to adjust to their new condition.^2–4^ Amputees can experience feelings of social discomfort,^5^ may avoid particular social scenarios^6,7^ and can exhibit symptoms of depression.^8–12^ In fact, concern about body image has been directly linked to depression in amputees.^3,4,10^ The appearance or aesthetics of a prosthetic limb is important to amputees^13–18^ and can influence their opinion or acceptance of the prosthesis.^19–21^ Unsatisfactory prosthetic aesthetics are likely to negatively impact how amputees view their body. Therefore, improving prosthesis aesthetics may have a positive impact on an individual’s body image and consequently improve their psychological well-being. Prosthesis aesthetics are intrinsically linked to the cosmetic cover (cosmesis) fitted over the mechanical limb. Consequently, feedback from amputees about their satisfaction with the cosmesis and the importance they place on attributes of the design is essential to identify areas which require design improvement and to focus future cosmesis research.

Questionnaires developed for use with lower limb amputees that have been validated and are readily accessible include Trinity Amputation and Prosthesis Experience Scales (TAPES),^22^ Prosthesis Evaluation Questionnaire (PEQ)^23^ and the Houghton Scale.^24^ Typical uses of the questionnaires include studies which have reported the functional outcomes of prosthesis use, amputee quality of life or level of pain experienced.^15,18,25–29^ TAPES and PEQ include three questions that directly relate to the cosmesis of the prosthesis, while the Houghton Scale has no cosmesis-related questions. However, TAPES and PEQ do not focus on cosmesis satisfaction in detail. They do not clearly phrase questions about the cosmesis only, separate from the mechanical limb and cosmesis combined, or ask questions about the importance users place on the cosmesis features. Therefore, in order to ascertain relevant information that can be used to gauge user opinion on current cosmesis products and guide cosmesis design improvements, a specific cosmesis questionnaire needed to be developed. Amputee questionnaire development is prevalent in the literature: many studies choose to develop their own questionnaire, rather than use a pre-validated one available in the literature, to ensure they collect the information they require.^13,15–17,19,21,30–34^

Accordingly, this article presents the development of a questionnaire to establish the satisfaction of lower limb amputees with their cosmeses and what they consider to be important features. The results of the questionnaire, issued to a sample of amputees in the United Kingdom, are reported, and the implications for future improvement in cosmesis design are discussed.

## Methods

### Questionnaire

A questionnaire, provided in supplementary Appendix 1, was developed to investigate amputee satisfaction with cosmeses and the importance of cosmesis design features. This was conducted in consultation with a group of UK stakeholders, including prosthetic manufacturers, clinicians and amputees. From initial stakeholder interviews, a list of possible cosmesis features was created from their suggestions and supplemented by literature sources. After reviewing the list, the researchers and project industry partners (Chas A Blatchford Ltd and Pace Rehabilitation Ltd) selected the cosmesis features that would help inform the direction and methodology of the larger research project (Engineering and Physical Sciences Research Council (EPSRC) EP/I000577/1: Customisation of Cosmetic Covers for Artificial Limbs. Nine relevant cosmesis features were identified for examination in the questionnaire and are listed in Table 1.

**Table 1.** List of cosmesis features examined in the questionnaire.

Feature subgroup	Cosmesis feature
Aesthetic	Colour
	Shape
	Touch/feel
Dynamics	The fit under clothes
	Lifelike/natural bending of the cosmesis
	Influence on prosthetic joint movement
Maintenance	Waterproof quality
	Ability to keep clean
	Durability

Demographic information was also collected and information about the type of cosmesis and the typical cosmesis lifespan. An open-ended question was available to record any details the participant thought were relevant to cosmesis that had not been raised in the questionnaire. In order to assess that the respondents understood the question meaning, each of the satisfaction questions about the 9 features was repeated using alternative wording, providing 18 possible responses in total to question number 12. The nine paired responses were then available for inter-wording reliability analysis. The words analysed are highlighted in bold in supplementary Appendix 1. Participants who did not wear a cosmesis were requested to only answer the importance question. The questionnaire was pilot tested to ensure that the language was understandable and no key cosmesis features had been omitted. Minor changes were made to the layout and question language prior to issue. All of this contributed evidence to support initial content and face validity. Strathclyde University ethical approval was granted for the study.

### Participants

The questionnaire was targeted at lower limb amputees of all levels and aetiologies in the age range 18–70 years with the cognitive ability to understand and complete the questionnaire. The questionnaire was issued by post to 296 members of the Murray Foundation, a registered charity in Scotland. An additional 100 questionnaires were issued via prosthetic appointments in England provided by the project partners.

### Data analysis

All questionnaire responses were collected by post. The response data were inputted manually to SPSS version 20, and all analysis was performed on either SPSS V20 or Minitab V16. Due to the low number of respondents in some demographic categories, several variables were re-coded. The details are provided in supplementary Appendix 1. The demographics were frequency counted and reported as a percentage of the sample. The continuous scale satisfaction scores in question 12 were converted to an integer when input to SPSS (0–10; calculated to the nearest centimetre). A score of 5 was considered to be a neutral opinion, while a score less than 5 was considered dissatisfied. The satisfaction scores taken from the first nine sections of question 12 were frequency counted, and the percentage of respondents reporting a satisfaction score of 5 or less was calculated for each feature. Important features were given a point score of 3, 2 or 1 corresponding to the most important feature to third most important, respectively, and the scores were summed for each feature. The importance ranking of each feature was calculated as a percentage of the maximum possible score of 336 (feature rated most important by the 112 respondents who completed the questions correctly).

In the initial consultations with stakeholders, preconceived opinions were repeatedly voiced by clinicians. These were anecdotal generalisations without evidence to support them. Therefore, directional relationships were identified from the anecdotal opinions, in the form of hypotheses. Evidence of statistically significant associations between demographic features was tested using chi-square or Fisher’s exact tests where appropriate. One-tailed Fisher’s exact tests were used to allow for directional conclusions to be drawn from any significant associations based on the aforementioned hypotheses. Two-tailed Mann–Whitney tests were used to test for evidence of a significant difference between dichotomous demographic features or groups (transtibial, transfemoral) and cosmesis satisfaction or importance rating. Z-tests for proportion were also used when appropriate. A significance level of *α* = 0.05 was used for all analyses. Inter-wording reliability (equivalence of alternative wording) in question 12 was assessed using an inter-class correlation (ICC) and mixed Model (2.1) and subsequently, the standard error of measurement was calculated for each item in question 12.

## Results

### Demographics

A total of 153 responses were received (39% response rate). The sample was 69% male with 78% aged between 45 and 70 years. The limb amputation level was 67% transtibial, 27% transfemoral, 3% knee disarticulation and 3% other (hip disarticulation, partial foot). There was no statistically significant relationship between amputation level and either gender or age. Cause of amputation included trauma (33%), peripheral vascular disease (PVD) (18%), diabetes (15%), congenital defect (11%), cancer (9%) and other (14%). Cause of amputation was statistically significantly related to gender (*p* = 0.019). Amputations due to PVD and diabetes, while slightly more prevalent in males, were not significantly higher (*p* = 0.186 and 0.240, respectively); however, trauma amputation was significantly more prevalent in males (*p* = 0.039).

**Figure 1.**
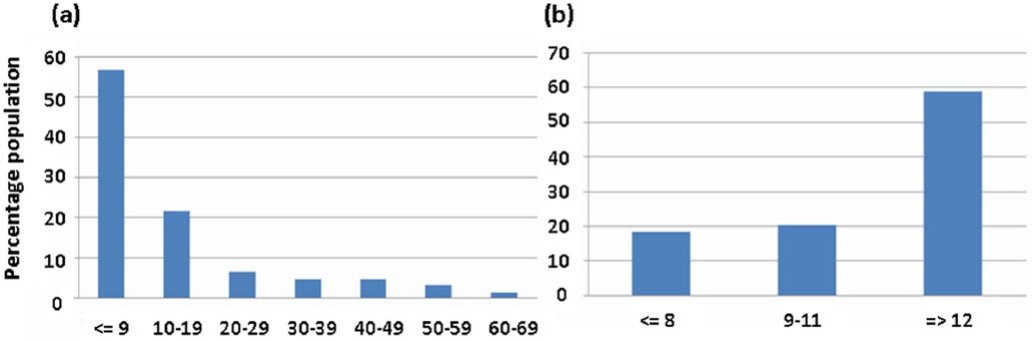
Prosthesis use reported in terms of (a) total years wearing a prosthesis and (b) hours per day wearing current prosthesis.

Prosthesis use is illustrated in Figure 1. When asked to assess their activity level, 15% of the amputees classed themselves as indoor-only walkers, 48% as limited outdoor walkers, 23% considered themselves active outdoor walkers and 7% very active sporting participants (7% missing data). The proportion of female active outdoor walkers was significantly greater than those who were male (*p* = 0.007). However, no statistically significant association was found between physical activity level and any of the following demographic features: age, amputation level, or how many hours per day the prosthesis was worn.

Of the respondents, 73% (*n* = 111) had a cosmesis fitted to their current prosthesis; 66% of the cosmeses were made from soft polyurethane foam and 33% from hard plastazote foam. In all, 73% had nylon stocking outer covers, 20% had prefabricated or custom silicone covers and 7% had other cover types (polyvinylchloride (PVC) or spray polyurethane coating). Wearing a cosmesis was significantly more common among 45- to 70-year-olds than 18- to 44-year-olds (*p* = 0.002), yet no significant relationship was found between wearing a cosmesis and the following demographic features: gender, amputation level and physical activity level. The use of a soft foam cosmesis was significantly more common for transfemoral than transtibial amputees (*p* = 0.001). For 37% of the cosmesis users, the cosmesis lifespan was typically 12 months or less, while the lifespan was greater than 12 months for 54% of the users (remaining percentage had missing data). A significantly larger proportion of females reported a short cosmesis lifespan (12 months or less) than males (*p* = 0.006). However, cosmesis lifespan was not statistically linked to any of the following demographic features: age, physical activity level, type of foam and type of outer cover.

### Cosmesis satisfaction

The ICC results when comparing question 12(a) through 12(i) with 12(j) through 12(r) are provided in Table 2. Values above 0.7 are considered satisfactory,^35^ while others^36^ suggest, for development research, values greater than 0.6 are acceptable. Therefore, these results were considered acceptable for the inter-wording reliability within the question, thus strengthening the psychometric properties of the instrument. Table 3 lists the percentage of cosmesis wearers who reported a neutral or dissatisfied opinion for each cosmesis feature (score of 5 or less).

**Table 2.** ICC results for inter-wording reliability and their 95% CI with SEM.

Satisfaction question topic	ICC	95% CI^a^	SEM
Colour	0.848	0.780–0.896	1.06
Shape	0.853	0.788–0.899	1.06
Touch/feel	0.784	0.692–0.850	1.36
The fit under clothes	0.821	0.746–0.875	1.07
Lifelike/natural bending of the cosmesis	0.870	0.811–0.911	1.08
Influence on prosthetic joint movement	0.682	0.557–0.776	1.55
Waterproof quality	0.897	0.847–0.931	0.81
Ability to keep clean	0.828	0.750–0.882	1.16
Durability	0.938	0.909–0.958	0.70

CI: confidence interval; SEM: standard error of measure; ICC: inter-class correlation.

a Each ICC was significant at *p* < 0.001.

**Table 3.** Percentage of cosmesis wearers reporting a neutral or dissatisfied opinion.

	Cosmesis feature	% of cosmesis wearers^a^
Aesthetic	Colour	59
	Shape	49
	Touch	57
Dynamics	The fit under clothes	45
	Lifelike/natural bending of the cosmesis	58
	Influence on prosthetic joint movement	43
Maintenance	Waterproof quality	61
	Ability to keep clean	64
	Durability	45

a Percentage of actual responses to each item – varying from 92 to 101 of the 111 wearers.

On average, females were less satisfied than males with the durability of their cosmesis (*p* = 0.016); however, males and females had similar satisfaction levels for all other cosmesis features. Younger amputees (18–44 years old) were on average less satisfied than older amputees (45–70 years old) with the feel of the cosmesis (*p* = 0.034) and its durability (*p* = 0.024). However, they had similar satisfaction levels for all other cosmesis features. Transfemoral amputees were on average less satisfied than transtibial amputees with the waterproof quality (*p* = 0.002) and durability (*p* = 0.029) of the cosmesis; however, they had similar satisfaction levels for the feel, shape, fit under clothes, influence on prosthetic movement and lifelike bending. On average, active walkers and limited outdoor walkers had similar satisfaction levels for the waterproof quality, influence on prosthetic movement, lifelike bending and cosmesis durability; however, active walkers were less satisfied (*p* = 0.028) with the cosmesis fit under clothes. On average, amputees with a nylon stocking–covered cosmesis were less satisfied than those with a silicone cosmesis with respect to the waterproof quality (*p* < 0.001) and the ability to keep clean (*p* = 0.003); however, satisfaction levels were similar for all other features.

### Important cosmesis features

The nine cosmesis features are listed in Table 4 in order of importance as determined by the importance ranking. The importance ranking was calculated as a percentage of the maximum possible score of 336. There was no statistically significant relationship between gender and the most important ranking of any of the following cosmesis features: colour match, shape match, durability and fit of clothes over the cosmesis. Similarly, there was no statistical relationship between amputation level and the first place ranking of any of the following cosmesis features: durability, free prosthetic movement and fit of clothes. There was no statistical evidence that proportionally more active walkers than indoor walkers ranked durability or free prosthetic movement as the most important feature.

**Table 4.** Importance ranking of cosmesis features, listed in order of importance.

Cosmesis feature	Importance (%)
Shape matched to sound limb	41
Free prosthetic movement under cosmesis	32
Natural fit of clothes over the cosmesis	31
Colour matched to skin tone	26
Durability	22
Lifelike/natural bending of the cosmesis	22
Waterproof quality	10
Touch/feel	8
Easy to clean surface	7

## Discussion

The questionnaire response rate (39%) is typical of amputee studies using self-return postal questionnaires and lies within the range reported in the literature (37%–81%).^17–22,28,29,33,37,38^ Given that PVD is the cause of over 90% of lower limb amputations in the developed world,^39^ it may be considered unusual that only 18% of the sample were PVD amputees, while 33% of the sample were trauma amputees. However, the amputee population recruited via the clinical services of Pace Rehabilitation Ltd is primarily trauma related. Therefore, this recruitment method could account for the larger percentage of trauma amputees in the sample.

Examples of some commonly held opinions identified in the stakeholder consultations include that women are more likely to wear a cosmesis than men, transtibial amputees are more likely to wear a cosmesis than transfemoral amputees and amputees with low physical activity level are more likely to be cosmesis wearers than those who are very active. In this sample, however, there was no evidence to support these opinions; no statistically significant relationship was found between using a cosmesis and either gender, amputation level or physical activity level. Similarly, there was no statistical evidence to support the view that the lifespan of the soft foam cosmesis is shorter than the hard foam cosmesis or that the cosmesis lifespan is linked to the activity level of the amputee. There is thus a lack of statistical evidence in support of the anecdotal viewpoint of some clinicians about the types of amputees who are likely to be cosmesis wearers.

The majority of the sample reported a neutral or dissatisfied opinion with five of the nine cosmesis features identified (Table 3). The finding indicates that amputee satisfaction is below the level that the prosthetic industry and clinical community would strive to attain in the design and fitting of cosmeses. In particular, 49%–59% of the respondents were neutral/dissatisfied with the three aesthetic features: colour, shape and touch/feel of the cosmesis. Considering the established link between prosthetic limb appearance and amputee opinion or acceptance,^19–21^ increasing amputee cosmesis satisfaction by improving cosmesis design is required to positively impact the psychological well-being of amputees.

The directional relationships tested using the satisfaction scores identified that on average, females were less satisfied than males with cosmesis durability. This is reinforced by the finding that a significantly larger proportion of females reported a short cosmesis lifespan than males. There was, however, no statistical evidence to support the anecdotal opinion that females were less satisfied than males with any other cosmesis feature, including the shape, the colour and the fit underneath clothing. Again, this contrasts with the view held by some clinicians that female amputees are less satisfied with the aesthetic qualities of the cosmesis than males.

Transfemoral amputees were on average less satisfied with cosmesis durability and waterproof quality than transtibial amputees. In this instance, the statistical evidence supports the opinion that transfemoral amputees are less satisfied than transtibial amputees; however, only with regard to these two features, the satisfaction levels are similar for all other features, including the influence of the cosmesis on prosthetic joint movement. This finding is of particular interest as the cosmesis can alter transfemoral amputee gait because the prosthetic knee joint is surrounded by the foam.^40,41^ While the clinician is aware of the impact of the foam cosmesis on the knee joint, perhaps this is less obvious to the transfemoral amputee as they typically do not have a point of reference with which to compare.

On average, amputees wearing a nylon stocking–covered cosmesis, the low-cost cosmesis provision in the United Kingdom, were less satisfied with the waterproof quality and the ability to keep it clean than those wearing a silicone cosmesis. However, satisfaction levels were similar for all other features, including the shape, colour and touch/feel of the cosmesis. This evidence is interesting given that the silicone cosmesis aims to achieve a high-quality aesthetic finish with limb-like realism, and they are considerably more expensive than the nylon stocking covers. Yet, the findings suggest that amputees have similar satisfaction levels with the lower cost option.

Questionnaire participants were requested to rank which three of the nine cosmesis features they considered most important. The ranking was limited to three choices in an attempt to reduce the questionnaire time and therefore improve the response quality.^42^ Furthermore, the questionnaire was intended to guide the future improvements of cosmesis design, and the authors and industry partners considered three features a manageable list to focus the research. The most important cosmesis feature identified by the sample was the shape match to the sound limb, followed by free prosthetic joint movement under the cosmesis and the natural fit of clothing over the cosmesis. The importance rankings identify areas of priority for research and development in the near future. In addition, the features with the highest percentage of dissatisfied/neutral amputee opinion (colour, waterproof quality and ability to keep clean) were not ranked with high importance. This demonstrates the need to question both satisfaction, to gauge approval and acceptance of current products, and importance, to extract the appropriate data to inform future cosmesis design.

Stakeholders held the following opinions: cosmesis shape, colour, durability and fit under clothes would be more important to females than males; cosmesis durability, free prosthetic joint movement and cosmesis fit under clothes would be more important to transfemoral than transtibial amputees and cosmesis durability and free prosthetic joint movement would be more important to active walkers than those limited to indoor walking. However, there was no statistical evidence to support any of these opinions. This highlights the potential value of using the information gathered in this study to test for evidence to support any common anecdotal opinions in the clinical community. It may be useful for clinicians to evaluate if they harbour any preconceptions and if this influences their clinical work.

Possible limitations of the study include the total number of respondents along with the effect this may have had on some demographic sub-categories thus limiting the ability to generalise the results. The instrument itself is at a developmental stage, and while some aspects of reliability and validity have been addressed in this article, outstanding work still exists in this area, and the authors actively encourage this work.

## Conclusion

The results indicate that current amputee satisfaction levels with their cosmesis are below what the medical device industry and clinical community would desire. The most important cosmesis features identified by the sample can be used to direct future cosmesis design research.
